# Multimorbidity patterns in high-need, high-cost elderly patients

**DOI:** 10.1371/journal.pone.0208875

**Published:** 2018-12-17

**Authors:** Alessandra Buja, Mirko Claus, Lucia Perin, Michele Rivera, Maria Chiara Corti, Francesco Avossa, Elena Schievano, Stefano Rigon, Roberto Toffanin, Vincenzo Baldo, Giovanna Boccuzzo

**Affiliations:** 1 Department of Cardiologic, Vascular, and Thoracic Sciences and Public Health, University of Padova, Padova, Italy; 2 Department of Statistical Sciences, University of Padova, Padova, Italy; 3 Department of Surgery, Oncology and Gastroenterology, University of Padova, Padova, Italy; 4 Veneto Regional Local health Unit, Venice, Italy; University of Alberta, CANADA

## Abstract

**Introduction:**

Patients with complex health care needs (PCHCN) are individuals who require numerous, costly care services and have been shown to place a heavy burden on health care resources. It has been argued that an important issue in providing value-based primary care concerns how to identify groups of patients with similar needs (who pose similar challenges) so that care teams and care delivery processes can be tailored to each patient subgroup. Our study aims to describe the most common chronic conditions and their combinations in a cohort of elderly PCHCN.

**Methods:**

We focused on a cohort of PCHCN residing in an area served by a local public health unit (the “Azienda ULSS4-Veneto”) and belonging to Resource Utilization Bands 4 and 5 according to the ACG System. For each patient we extracted Expanded Diagnosis Clusters, and combined them with information available from Rx-MGs diagnoses. For the present work we focused on 15 diseases/disorders, analyzing their combinations as dyads and triads. Latent class analysis was used to elucidate the patterns of the morbidities considered in the PCHCN.

**Results:**

Five disease clusters were identified: one concerned metabolic-ischemic heart diseases; one was labelled as neurological and mental disorders; one mainly comprised cardiac diseases such as congestive heart failure and atrial fibrillation; one was largely associated with respiratory conditions; and one involved neoplasms.

**Conclusions:**

Our study showed specific common associations between certain chronic diseases, shedding light on the patterns of multimorbidity often seen in PCHCN. Studying these patterns in more depth may help to better organize the intervention needed to deal with these patients.

## Introduction

Patients with complex health care needs (PCHCN) are individuals whose care requirements are numerous and costly, and they have been shown to place a heavy burden on health care resources [1]. This may be an over-simple definition, however, and efforts in the US to better classify PCHCN have shown that this group mainly consists of frail elderly people or individuals with multimorbidity. This term refers to the co-occurrence of multiple chronic or acute diseases without any one disease being taken for reference, whereas comorbidity is usually defined in relation to a given index condition [2]. According to Schellevis, comorbidity can also be classified by the relationship between the different diseases [3]: concurrent comorbidity defines the random coexistence of diseases; cluster comorbidity indicates statistically significant associations between diseases without a causal explanation; causal comorbidity describes disease clustering with a pathophysiological relationship between the different diseases (e.g. shared risk factors); and complicating comorbidity illustrates the case when one disease is caused by another, and cannot be explained without its precursor [3–4]. Multimorbidity poses new challenges to health care services, which have traditionally focused on one disease at a time. Several research methods have been used to shed light on the complexity of multimorbidity, based on the fundamental assumption that health outcomes in patients with multimorbidity are largely influenced by their single diseases with potential add-on effects of interactions between them [5]. There is evidence to suggest that chronic conditions form clusters [6], and a patient-by-patient measure of such clusters of morbidities is essential to both the funding of medical care and the planning of prevention and treatment services. In elderly patients, understanding the relationship between concomitant diseases may help us to develop strategies to improve clinical practice and prevention measures [7]. The disease clusters approach could also serve in prioritizing the development of new multimorbidity guidelines for the most common diseases and combinations thereof [8].

On the other hand, targeting patients on the basis of cost alone, without considering their personal characteristics and needs, might not properly identify those for whom an intervention for example of case -management would be most effective [9]. In fact, many unsuccessful programs enroll large percentages of people who are unlikely to benefit from the intervention. Simply being high-need/high-cost is not always enough stratification method. So there has been a growing interest in targeting the needs of these PCHCN populations, and those of populations at risk of joining their ranks with a view to designing tailored health care models capable of improving these patients’ health outcomes while containing the related costs [10]. It has been argued that an important factor in providing value-based primary care concerns how to identify groups of patients with similar needs (who pose similar challenges) so that care teams and care delivery processes can be tailored to different patient subgroups [11].

In a population of elderly PCHCN, our study aimed to identify their multiple chronic conditions (multimorbidity) and how they were combined (as dyads or triads) in an effort to provide a better epidemiological picture of this high-need clinical group.

## Materials and methods

### Context

The Italian NHS (National Health System) is a public system financed mainly by general taxation. It is grounded on fundamental values of universality, free access, freedom of choice, pluralism in provision, and equity. Regional authorities plan and organize health care facilities and activities through their regional health departments in accordance with a national health plan designed to assure an equitable provision of comprehensive care throughout the country. The regional authorities coordinate and control local health units (LHU), each of which is a separate geographically based public company delivering public health, promotion and community health services, primary care and hospital care, either with their own facilities and personnel or through outside contractors [12]. The Veneto Regional Health Service has 21 such LHUs serving a population of about five million. The LHU involved in the present study was the “Azienda ULSS4-Veneto”, which serves a population of about 190,000 in the province of Vicenza, in northeastern Italy.

The ACG System was implemented in the Veneto Region in 2012 as a tool for population risk stratification [13–15]. The ACG System is a method used internationally to characterize multimorbidity on the strength of routinely collected administrative data (e.g. hospitalization records, pharmaceutical prescriptions, access to emergency departments, prescriptions charge exemptions) gathered together using record linkage. It relies on an algorithm that starts from individual-level diagnoses and is based on the clinical judgement of likelihoods (persistence or recurrence over time, demand for specialist services, hospitalization, disability or decline in quality of life, expected need for and use of diagnostic or therapeutic procedures), then adjusted for age and gender, to group a population by 93 mutually-exclusive combinations of conditions, or ACG (Adjusted Clinical Groups) that represent clinically logical categories of patients expected to require similar levels of health care resources [16]. Based on usage of care resources, the ACG System automatically collapses the different ACG categories into six Resource Utilization Bands (RUBs), which are defined as follows: 0, nonuser or invalid diagnosis; 1 healthy user; 2 low morbidity; 3 moderate morbidity; 4 high morbidity; 5 very high morbidity [17]. The ACG database is owned by the single LHUs and the Veneto Region’s Epidemiological Services, which may provide researchers with anonymized data for study purposes.

The present study only concerned patients aged 65 years or more residing in the area served by the LHU “Azienda ULSS4-Veneto”, and characterized by complex health care needs (PCHCN patients), corresponding to RUBS 4 and 5. For each individual considered, we extracted EDC (Expanded Diagnosis Clusters), which coincide with the clinical diagnosis that the ACG system assigns to single patients by combining different information flows. To improve the sensitivity of our model, patients with chronic conditions were also identified by means of the information available from the Pharmacy (RX)-based Morbidity Marker Groups (Rx-MGs), and the clinical criteria used to assign medication to different morbidity groups. The Rx-MGs provide further methods for describing the particular morbidity profile of a given population and form the basis of the pharmacy-based predictive model [17]. A dichotomous variable was thus assigned to each chronic disease (1 if one of the EDCs or Rx-MGs were involved, 0 otherwise). Cases of neoplastic disease, Alzheimer’s disease, fibrillation and cerebrovascular disease were only discernible from the ECD codes, while cases of hyperlipidemia could only be obtained from the Rx-MGs.

Unfortunately, no standard exists for measuring multimorbidity, so the choice of morbidities to consider is always inevitably rather subjective and depends on the data available [18]. This study focused on a subset of conditions including: cancer, congestive heart failure, ischemic heart disease, high blood pressure (HBP), atrial fibrillation, hyperlipidemia, cerebrovascular disease, Alzheimer’s disease, depression, asthma/bronchitis, diabetes, chronic obstructive pulmonary disease, osteoporosis, hypothyroidism, and chronic renal disease.

In addition to the disease-specific variables, patients’ age was also considered, grouped into the following age brackets: “65–69”, “70–74”, “75–79”, “80–84”, “85+”.

### Statistical methods

Frequencies and percentages were used for the descriptive analysis. A first bubble graph was constructed to show the estimated proportions of patients with each dyad of diseases–the larger the bubble, the higher the observed prevalence of a given dyad. Then a second bubble graph was used to indicate the magnitude of the association, measured with the chi^2^ statistic.

The prevalence was calculated for the most common disease dyads (for which the observed prevalence was at least 20%), and, the association between the diseases comprising each dyad was estimated by means of odds ratios and their 95% confidence intervals. To see whether these prevalences were greater than might be expected, the observed-to-expected prevalence ratio were also calculated (given the prevalence of each disease in the population, the expected prevalence of a given disease dyad is estimated by assuming that the two diseases occur independently). The same was done for the most common disease triads (and we report those with an observed prevalence of at least 10%). The most common triads were counted in terms of absolute frequency, and per 100 respondents. The observed-to-expected prevalence ratios are reported with 95% confidence intervals.

A cluster analysis was undertaken on the variables to identify clinically meaningful groups of chronic diseases. A key concept in clustering is dissimilarity, and the problem is how to measure it properly: Jaccard’s distance was adopted here because the variables in question are dichotomous (the presence or absence of a given disease). It was appropriate to analyze clusters of variables with the hierarchical clustering approach because many chronic diseases share the same underlying genetic, environmental or behavioral risk factors. The complete linkage criterion was used to measure the distances between clusters because it led to the best result. An advantage of hierarchical clustering on variables is that the outcome can easily be represented by means of a dendrogram.

An exploratory latent class analysis (LCA—a tool appropriate for analyzing categorical data) was undertaken to classify patients in a number K of classes, which had to be defined a-priori. The number was chosen using the Bayesian information criterion (BIC). Ten different models were delineated, characterized by an increasing number of classes (from one to ten), and the model that gave rise to the smallest BIC index was adopted. The model with five latent classes was consequently chosen, which led to a BIC of 40,488.50.

### Ethical considerations

The data analysis was performed on anonymized aggregate data with no chance of individuals being identifiable. The study complied with the Declaration of Helsinki and with Italian Law n. 196/2003 on the protection of personal data. The recent resolution n. 85/2012 of the Italian Guarantor for the Protection of Personal Data also confirmed the allowability of processing personal data for medical, biomedical and epidemiological research, and that data concerning health status may be used in aggregate form in scientific studies. Permission to use unidentifiable individual data extracted from administrative databases was granted by the ex-ULSS 4, Veneto Region.

## Results

Our analysis was conducted on data drawn from a population of 185,887, selecting 39,643 individuals aged 65 years old or more, including 2,691 who were PCHCN on the grounds of the ACG: 2,250 (84%) were RUB 4, and 441 (16%) were RUB 5.

**Fig 1:**
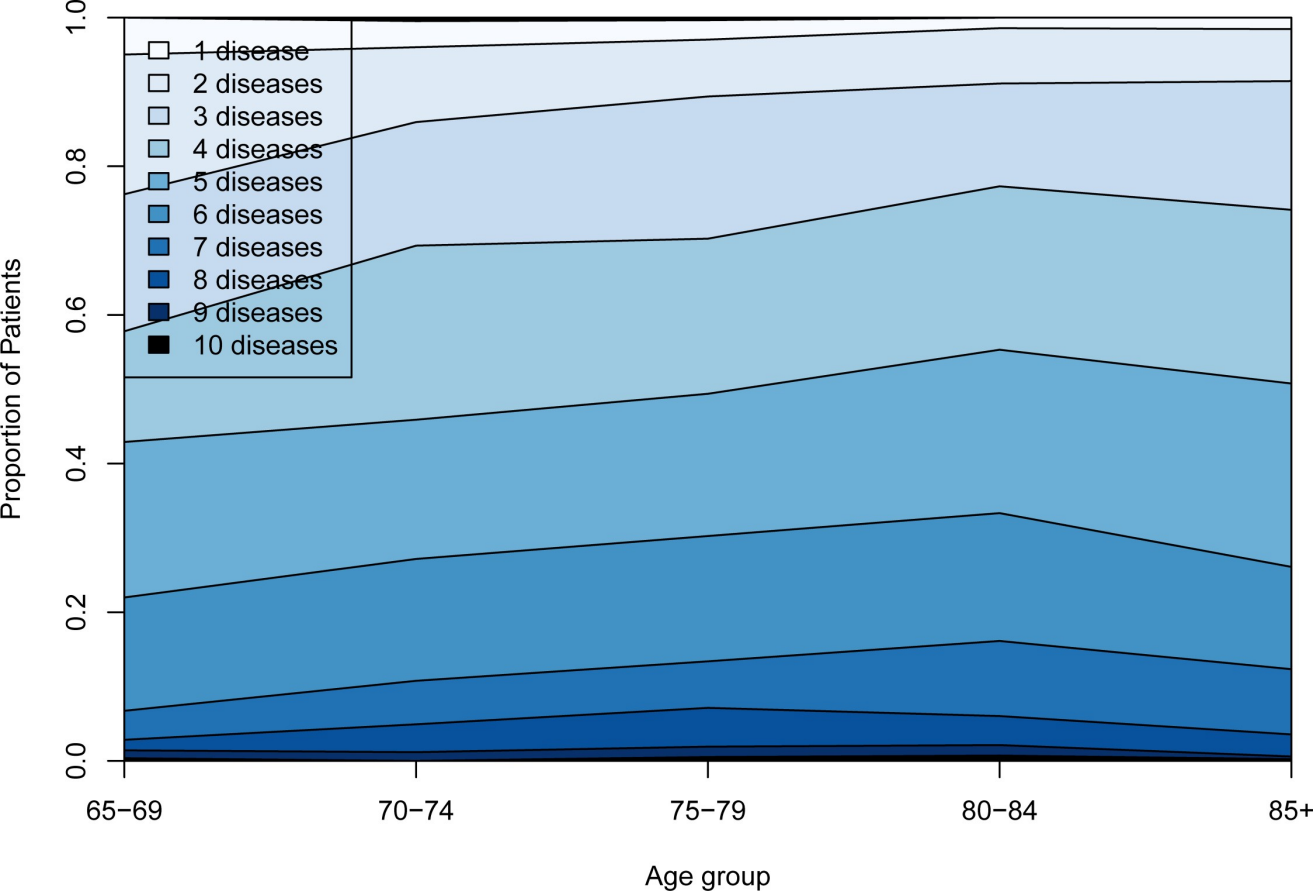
Number of chronic disorders by age group.

Table 1 provides demographic details of the study population, the proportion of those with multimorbidity and the proportion with multimorbidity. Among the PCHCN, the number of diseases involved and the proportion of people with multimorbidity did not increase substantially with age (Fig 1): at 65–69 years old, 95% of the sample already had more than one morbidity.

**Table 1:** Morbidity and multimorbidity in PCHCN.

** **	**N (%)**	**Mean number of morbidities**	**Percentage (95% CI) with multimorbidity**	**Percentage (95% CI) with multimorbidity involving both physical not neurological and neurological/mental health****°**
**ALL PATIENTS**	2691 (100.00%)	4.55 (1.72)	97.43% (96.84–98.03)	56.08% (54.20–57.95)
**SEX**				
**Female**	1384 (48.57%)	4.54 (1.67)	97.32% (96.71–97.93)	60.69% (58.12–63.27)
**Male**	1307 (51.43%)	4.55 (1.77)	97.54% (96.96–98.13)	51.19% (48.48–53.90)
**AGE**				
**65–69**	282 (10.48%)	4.05 (1.78)	95.04% (94.21–95.86)	40.78% (35.04–46.52)
**70–74**	427 (15.86%)	4.41 (1.75)	96.01% (95.28–96.76)	45.20% (40.48–49.92)
**75–79**	575 (21.37%)	4.59 (1.80)	97.04% (96.40–97.68)	52.52% (48.44–56.60)
**80–84**	564 (20.96%)	4.81 (1.72)	98.58% (98.13–99.03)	59.57% (55.52–63.62)
**85+**	843 (31.33%)	4.58 (1.58)	98.46% (97.99–98.92)	66.79% (63.61–69.96)
**NUMBER OF DISORDERS**				
	**N (%)**	**Cumulative frequencies**		**Percentage (95% CI) with multimorbidity involving both physical not neurological and neurological/mental health°**
**0**	4 (0.15%)	0.15%		-
**1**	65 (2.42%)	2.57%		-
**2**	241 (8.96%)	11.53%		31.54% (25.67–37.40)
**3**	457 (16.98%)	28.51%		46.17% (41.60–50.74)
**4**	583 (21.66%)	50.17%		51.80% (47.74–55.86)
**5**	581 (21.59%)	71.76%		61.45% (57.49–65.40)
**6**	423 (15.72%)	87.48%		69.03% (64.62–73.44)
**7**	203 (7.54%)	95.02%		77.34% (71.58–83.10)
**8**	97 (3.60%)	98.62%		84.54% (77.34–91.73)
**9**	27 (1.00%)	99.62%		81.48% (66.83–96.13)
**10**	10 (0.37%)	100.0%		100.00% (—)

°Morbidities involving the physical health: cancer, coronary heart disease, diabetes mellitus, high blood pressure, heart failure, chronic obstructive pulmonary disease, chronic kidney disease, atrial fibrillation, asthma, osteoporosis, hypothyroidism, hyperlipidemia. Morbidities involving the neurological/mental health: Alzheimer’s disease, cerebrovascular disease, depressive disorder.

**Fig 2:**
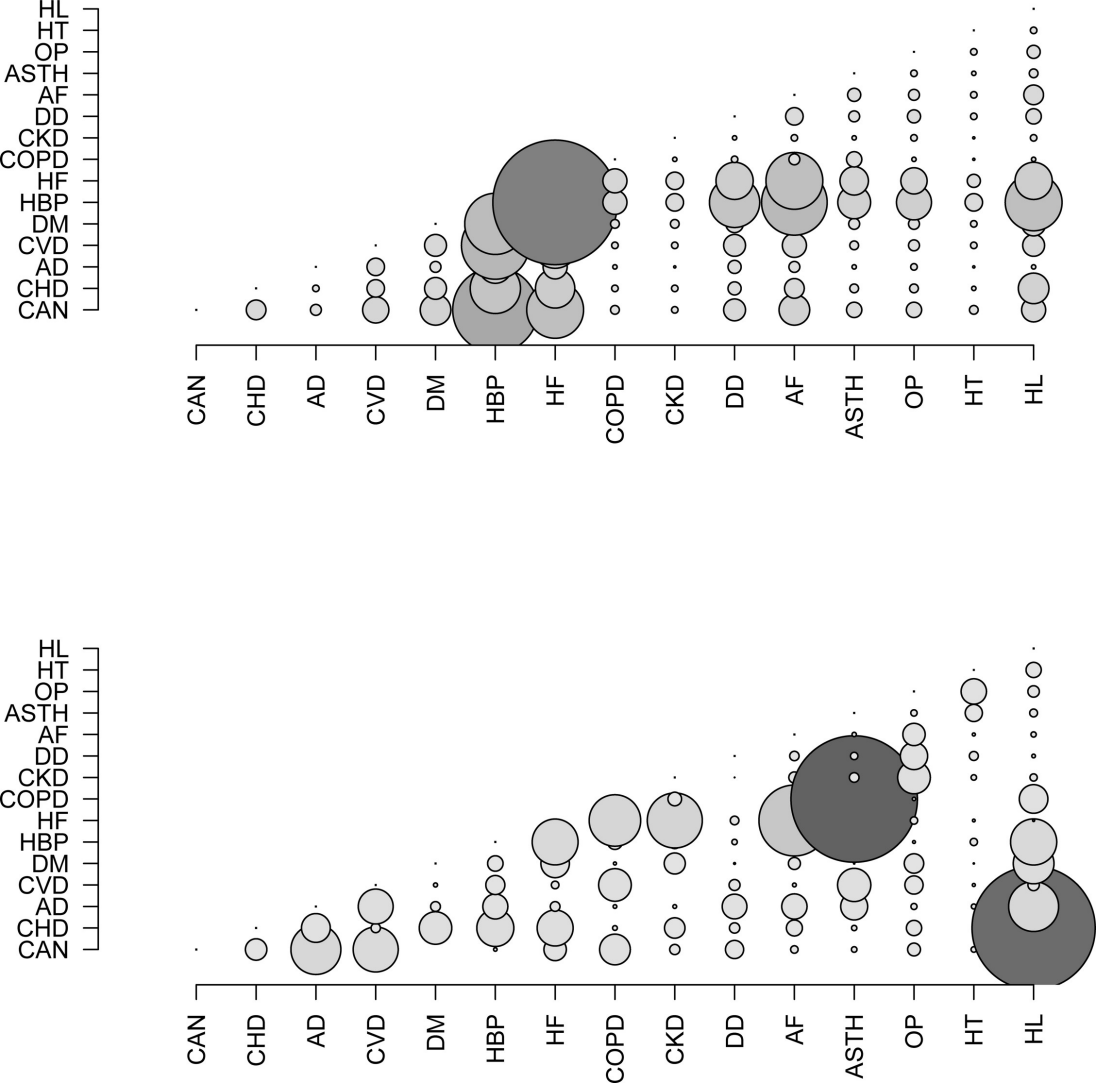
Bubble chart. (a) Bubble chart for the prevalences observed. A larger bubble indicates a higher observed prevalence of the dyad. (b) Bubble chart for the associations between chronic diseases, measured with the chi^2^ statistic. A larger bubble indicates a stronger association between the diseases. CAN = cancer; CHD = coronary heart disease; AD = Alzheimer’s disease; CVD = cerebrovascular disease; DM = diabetes mellitus; HBP = high blood pressure; HF = heart failure; COPD = chronic obstructive pulmonary disease; CKD = chronic kidney disease; DD = depressive disorder; AF = atrial fibrillation; ASTH = asthma; OP = osteoporosis; HT = hypothyroidism; HL = hyperlipidemia.

Fig 2A shows the observed prevalence of the disease dyads in PCHCN. As hypertension and congestive heart failure both have a high prevalence (88% and 63%, respectively), this dyad was found in 57% of patients.

Fig 2B shows the observed power of the associations between disease dyads among these patients. The most powerful associations were found between ischemic heart disease and hyperlipidemia, and between chronic obstructive pulmonary disease and asthma.

Tables 2 and 3 show the most common disease dyads before (Table 2) and after (Table 3) excluding hypertension and congestive heart failure, the latter in an effort to identify other interesting dyads among the less common diseases.

**Table 2:** Observed and expected prevalence (assuming the diseases occur independently, and based on the prevalence of each disease in the population) of all disease dyads with a prevalence higher than 20%. Dyads with statistically significant odds ratio are highlighted in bold.

DISEASE DYADS		Prevalence / 100				
		Observed	Expected	O/E	Odds ratio	Confidence interval
**HBP**	**HF**	57.41	55.12	1.04	2.40	**(1.90, 3.03)**
**CVD**	**HBP**	30.66	29.71	1.03	1.52	**(1.17, 1.97)**
**HBP**	**AF**	30.06	29.03	1.04	1.59	**(1.22, 2.08)**
**DM**	**HBP**	28.02	27.27	1.03	1.41	**(1.08, 1.84)**
**HBP**	**HL**	25.86	23.75	1.09	3.88	**(2.67, 5.63)**
**HF**	**AF**	25.83	20.77	1.24	2.89	**(2.40, 3.47)**
**HBP**	DD	23.23	22.96	1.01	1.14	(0.87, 1.49)
**CHD**	**HBP**	23.08	21.43	1.08	2.90	**(2.03, 4.14)**
**DM**	**HF**	21.52	19.51	1.10	1.51	**(1.27, 1.79)**

CAN = cancer; CHD = coronary heart disease; AD = Alzheimer’s disease; CVD = cerebrovascular disease; DM = diabetes mellitus; HBP = high blood pressure; HF = heart failure; COPD = chronic obstructive pulmonary disease; CKD = chronic kidney disease; DD = depressive disorder; AF = atrial fibrillation; ASTH = asthma; OP = osteoporosis; HT = hypothyroidism; HL = hyperlipidemia.

**Table 3:** Observed and expected prevalence (assuming the diseases occur independently, and based on the prevalence of each disease in the population) of disease dyads with a prevalence higher than 20%, after excluding hypertension and congestive heart failure.

DISEASE DYADS		Prevalence / 100				
		Observed	Expected	O/E		Odds ratio	Confidence interval
**CHD**	**HL**	13.97	6.60	2.12	6.39	**(5.27, 7.75)**
**DM**	**HL**	11.04	8.40	1.31	1.82	**(1.52, 2.17)**
**CHD**	**DM**	9.62	7.58	1.27	1.64	**(1.37, 1.97)**
**CHD**	**AF**	9.10	8.07	1.13	1.28	**(1.07, 1.54)**
**AD**	**CVD**	8.18	6.16	1.33	1.78	**(1.46, 2.17)**
**COPD**	**ASTH**	7.10	2.14	3.32	9.94	**(7.76, 12.73)**
**DD**	**OP**	6.28	4.81	1.31	1.61	**(1.30, 1.98)**
**AD**	**DP**	6.09	4.76	1.28	1.55	**(1.25, 1.91)**

CAN = cancer; CHD = coronary heart disease; AD = Alzheimer’s disease; CVD = cerebrovascular disease; DM = diabetes mellitus; HBP = high blood pressure; HF = heart failure; COPD = chronic obstructive pulmonary disease; CKD = chronic kidney disease; DD = depressive disorder; AF = atrial fibrillation; ASTH = asthma; OP = osteoporosis; HT = hypothyroidism; HL = hyperlipidemia.

Table 4 shows disease triads for which the observed prevalence was at least 10%, in declining order. The hypertension, congestive heart failure and fibrillation triad, and the ischemic heart disease, hypertension and hyperlipidemia triad revealed the greatest differences between the expected prevalence (assuming the diseases occurred independently of one another) and the observed prevalence. Other triads revealed a significantly higher observed than expected prevalence too, demonstrating that disease triads are associated in their occurrence, even if the analysis cannot clarify how the diseases are related.

**Table 4:** Observed and expected prevalence (assuming the diseases occur independently, and based on the prevalence of each disease in the population) of disease triads with an observed prevalence higher than 10%. Each row concerns one disease triad, and each chronic condition included in the triadi s marked with an (X). Rows with a statistically significant difference between the observed and expected prevalences are highlighted in bold.

DISEASE TRIADS															Prevalence / 100			
CAN	CHD	AD	CVD	DM	HBP	HF	COPD	CKD	DD	AF	ASTH	HT	OP	HL	O	E	O/E	CI
X					X	X									24.19	24.42	0.99	(0.90, 1.09)
					**X**	**X**				**X**					**23.86**	**18.23**	**1.31**	**(1.18, 1.45)**
				**X**	**X**	**X**									**20.07**	**17.13**	**1.17**	**(1.05, 1.31)**
			X		X	X									19.36	18.66	1.04	(0.93, 1.16)
	**X**				**X**	**X**									**16.98**	**13.46**	**1.26**	**(1.11, 1.43)**
					X	X								X	16.39	14.91	1.10	(0.97, 1.24)
					X	X			X						15.79	14.42	1.10	(0.96, 1.24)
	**X**				**X**									**X**	**13.79**	**5.80**	**2.38**	**(1.99, 2.84)**
X					X					X					12.86	12.86	1.00	(0.87, 1.15)
X				X	X										12.00	12.08	0.99	(0.86, 1.15)
					**X**	**X**					**X**				**11.71**	**9.12**	**1.28**	**(1.10, 1.50)**
					X	X							X		11.04	10.14	1.09	(0.93, 1.27)
**X**						**X**				**X**					**11.00**	**9.20**	**1.20**	**(1.02, 1.40)**
X					X									X	10.74	10.52	1.02	(0.87, 1.19)
				**X**	**X**									**X**	**10.59**	**7.38**	**1.43**	**(1.21, 1.71)**
			X		X					X					10.52	9.83	1.07	(0.91, 1.25)

CAN = cancer; CHD = coronary heart disease; AD = Alzheimer’s disease; CVD = cerebrovascular disease; DM = diabetes mellitus; HBP = high blood pressure; HF = heart failure; COPD = chronic obstructive pulmonary disease; CKD = chronic kidney disease; DD = depressive disorder; AF = atrial fibrillation; ASTH = asthma; OP = osteoporosis; HT = hypothyroidism; HL = hyperlipidemia.

**Fig 3:**
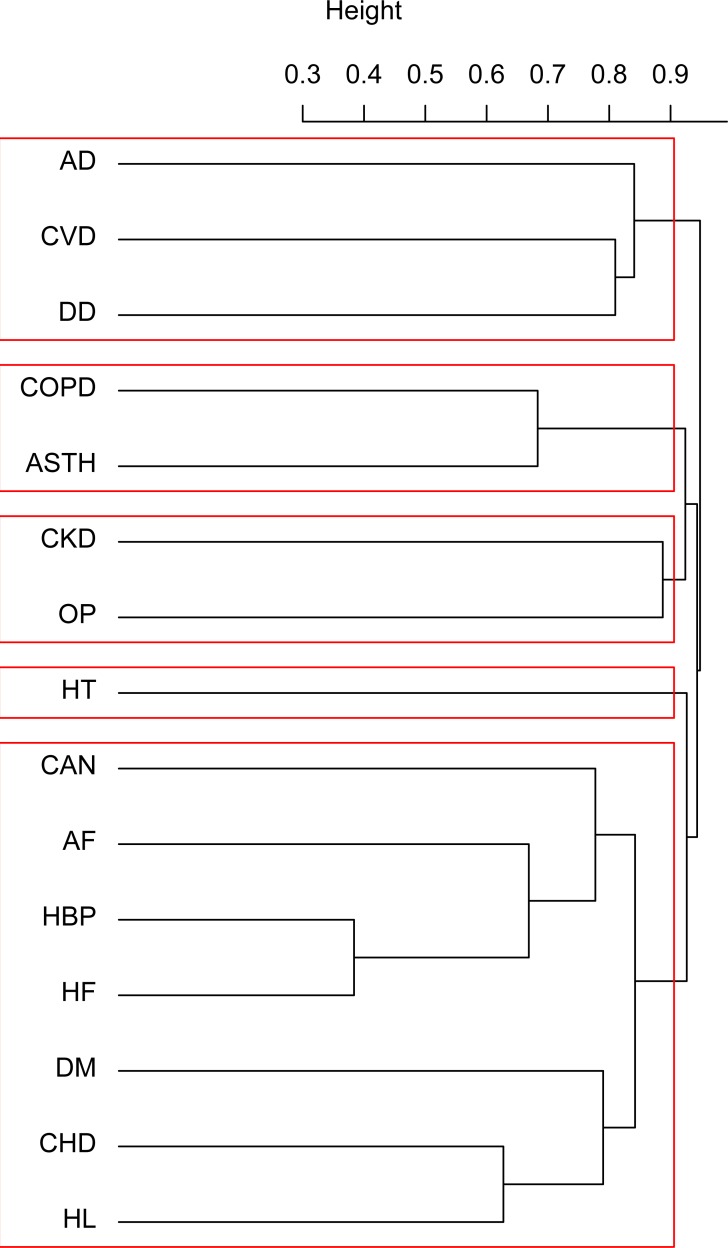
Dendrogram for hierarchical cluster analysis by disease. CAN = cancer; CHD = coronary heart disease; AD = Alzheimer’s disease; CVD = cerebrovascular disease; DM = diabetes mellitus; HBP = high blood pressure; HF = heart failure; COPD = chronic obstructive pulmonary disease; CKD = chronic kidney disease; DD = depressive disorder; AF = atrial fibrillation; ASTH = asthma; OP = osteoporosis; HT = hypothyroidism; HL = hyperlipidemia.

The result of hierarchical clustering on the variables is shown in a dendrogram (Fig 3). Five clusters are detectable, two of them clearly distinct (one comprising Alzheimer’s disease, cerebrovascular disease and depression, the other including chronic obstructive pulmonary disease and asthma). A larger cluster contains ischemic heart disease, diabetes, hypertension, congestive heart failure, fibrillation and neoplasms. Chronic renal failure and osteoporosis also form a cluster, while hypothyroidism seems to have no strong association with any of the other diseases considered here.

Table 5 shows the outcome of the model, with the class conditional probabilities for each disease. Ischemic heart disease, diabetes, hypertension and hyperlipidemia seem to be prevalent in the first class (which could be labelled as *Cardiovascular disease*), while the second is characterized by higher probabilities of Alzheimer’s disease, cerebrovascular disease and depression (and could be called *Neurological and mental illness*). Congestive heart failure and fibrillation have higher class-conditional probabilities related to Class 3 (*Cardiac disease*), and Class 4 includes the majority of patients with asthma and chronic obstructive pulmonary disease (the two *Respiratory diseases* considered here). The chronic diseases in Class 4 have very low class-conditional probabilities by comparison with the other classes. Patients with neoplasms are only prevalent in the fifth and last class (which was consequently labelled as *Cancer)*. Hypertension and congestive heart failure clearly have high class-conditional probabilities for more than one class, since they are the diseases with the highest prevalence in our dataset. On the other hand, chronic renal failure, osteoporosis and hypothyroidism feature low class-conditional probabilities for any of the five classes, confirming the weak association between these diseases and the other conditions considered here. Generally speaking, LCA produced results consistent with the cluster analysis on the variables.

**Table 5:** Class-conditional probabilities of each disease estimated with the five latent classes model. The higher class-conditional probabilities are highlighted in bold.

Disease	Latent classes				
	Class 1 *(cardiovascular)*	Class 2 *(Neurological and mental illness)*	Class 3 *(cardiac)*	Class 4 *(respiratory)*	Class 5 *(cancer)*
% of respondents in each class	22.4% (603)	19.1% (514)	30.2% (813)	10.3% (277)	18.0% (484)
**CAN**	41	17	**50**	28	**78**
**CHD**	**64**	8	22	21	8
**AD**	5	**46**	14	16	8
**CVD**	35	**61**	30	17	16
**DM**	**44**	22	33	30	26
**HBP**	**100**	82	**95**	84	75
**HF**	**67**	41	**99**	**92**	20
**COPD**	4	4	4	**100**	5
**CKD**	11	2	16	12	3
**DD**	26	**34**	25	28	18
**AF**	34	23	**53**	37	16
**ASTH**	12	4	15	**70**	12
**OP**	20	16	19	19	19
**HT**	12	7	8	9	12
**HL**	**99**	10	4	14	12

CAN = cancer; CHD = coronary heart disease; AD = Alzheimer’s disease; CVD = cerebrovascular disease; DM = diabetes mellitus; HBP = high blood pressure; HF = heart failure; COPD = chronic obstructive pulmonary disease; CKD = chronic kidney disease; DD = depressive disorder; AF = atrial fibrillation; ASTH = asthma; OP = osteoporosis; HT = hypothyroidism; HL = hyperlipidemia.

## Discussion and conclusions

Our study found consistent associations and sheds light on the patterns of multimorbidity identifiable using different analytical methods in a cohort of PCHCN. Our data revealed that nearly 90% of elderly PCHCN have at least two diseases, and more than one in three are at least 85 years old, thus confirming how multimorbidity as an important contributor to the complexity of health care in the late stages of life. Our data also underscore how PCHCN differ from the general elderly population, since it has been estimated that a total of 67% of Medicare beneficiaries have multimorbidity [19]. A greater analytical detail concerning multimorbidity in the elderly could be useful for planning treatment and prevention, and for both system-based and patient-centered policies. In fact, the clinical utility of any intervention can be improved by identifying particular combinations that might warrant an alternative diagnostic or therapeutic approach. Clinicians are sometimes uncertain about how to balance of benefit and harm of treatments for people with multimorbidity because the available evidence on treatment options is largely based on trials of interventions for single conditions, from which people with multimorbidity are often excluded [20]. The efficacy of more integrated approaches to the treatment of multimorbidity should be investigated initially in patients with specific disease combinations. Using a validated measure of multimorbidity, especially if it can characterize different aspects of this condition, could help outpatient clinicians and discharging physicians to orient patients towards appropriate treatment programs and primary care services (thereby improving case management, care coordination and the exploitation of a multidisciplinary team’s skills). The present study also showed that mental health impairments increase with age: if two in three PCHCN suffer from mental disorders (as well as physical health issues), this complicates their management. Studies have demonstrated that patients with mental health conditions are at risk of receiving suboptimal care for their hypertension, diabetes, heart failure, arthritis, and chronic obstructive pulmonary disease [21–23].

Our analysis confirms that chronic conditions tend to form clusters. For example, cardiovascular conditions cluster with certain metabolic disorders, as already reported elsewhere [4,24]. This association has a well-defined pathological mechanism and could be interpreted as an instance of causal comorbidity. Despite this evidence of clustering, guidelines on the treatment of chronic disease frequently fail to consider the issue of multimorbidity. It would seem imperative to revise guidelines on cardiovascular ischemic disease, for instance, to better address the most common associated morbidities. As guidelines drive care in certain settings, they may also provide the basis for assessing quality of care, so their failure to consider multimorbidity can have far-reaching, adverse implications. For example, Boyd et al. examined the applicability of US clinical guidelines to older individuals with several comorbid diseases in 2005. The study reviewed clinical guidelines for nine chronic conditions to ascertain whether they adequately addressed the care needs of older patients with multiple comorbid diseases. The authors found that clinical guidelines rarely addressed comorbidity, and adherence to guideline recommendations in caring for an older person with multimorbidity would often lead to complex and sometimes contradictory drug and self-care regimes [25]. In fact guideline-driven medication is taken in addition to usual prescriptions and over-the-counter drugs for conditions such as allergies, pain, dyspepsia, and insomnia. Viewing disease-specific medication guidelines from this perspective raises the question of whether what is good for the disease is always best for the patient [26]. Guidelines that take multimorbidity interrelatedness into account are also likely to be particularly beneficial to the geriatric community, because it is widely accepted that patients and health care providers need to simplify and prioritize treatment options in the case of multiple chronic conditions [27]. An example of one such effort is the 2012 consensus report jointly published by the American Geriatrics Society and the American Diabetes Association, which provides recommendations on the treatment of diabetes in the context of geriatric conditions such as dementia, functional impairment, and urinary incontinence [28]. The UK guidelines include strategies to adopt in an approach to care that takes multimorbidity into account. They suggest examining the benefits and risks of following the recommendations for single health conditions and improving quality of life by reducing the burden of treatments, adverse events and unplanned care, and by improving the coordination of care across services [29].

There is evidence to suggest that novel models of health care, such as medical care at home for cardiovascular patients, are needed to provide high-quality, efficient and effective care for our growing population of PCHCN [30].

Our cluster dendrogram shows that cancer contributed most strongly to the cardiovascular cluster. This intriguing finding might be explained by some well-known pathological mechanisms that these conditions have in common. Cardiovascular disease and cancer share a number of risk factors, such as smoking, obesity, a Western diet, and a sedentary lifestyle. A better understanding of the impact of multimorbidity and its management on cancer treatment, and vice versa, is also much needed. In the sphere of cancer prevention procedures, for instance, it may be that patients with multimorbidity have a cancer diagnosed earlier because they are more frequently in contact with medical services, or later because their physicians have been focusing only on their comorbid conditions [31].

In our patients in Class 2 (Neurological and mental illness) there was a higher conditional probability of depression, cerebrovascular disorders and dementia. Other authors have suggested an association between anxiety, depression and somatic symptoms that we are unable to fully support [24]. The strong association between depression, other cerebrovascular conditions and dementia might be due to the overlap between the psychological and neurological domains, and related to altered mood regulating circuits secondary to vascular problems [4]. A large body of literature has shown that: depression is common among the elderly, and associated with poor cognitive function [32]; a history of depression may confer a higher risk of developing Alzheimer’s disease, and depression could be considered an independent risk factor for this disease [33].

Overall, our findings confirm a high prevalence of multimorbidity among PCHCN, and point to the need for a patient-focused rather than a disease-focused management. Care plans designed and implemented to suit the individual needs of a given patient are of the utmost importance in order to manage and prioritize their multimorbidity. The implications downstream include a more efficient patient management, and better health outcomes for patients with complex multimorbidities [34]. Recent recommendations on patients with multimorbidity have focused more on general rather than disease-specific health outcomes, and on cognitive assessments, non-pharmacological treatments, and minimizing the burden of treatment for both patients and their caregivers, under the coordinated care of multidisciplinary teams [35]. The most advanced effort to address the complex health needs of elderly patients comes from the American Geriatrics Society (AGS), which convened an expert panel with complementary expertise on the relevant topics along with a special interest in older adults with multimorbidity [36]. A recent systematic review nonetheless found that it is still difficult to improve outcomes for people with multiple conditions and suggested that intervention designed to target specific risk factors, or focusing on difficulties people experience with daily functioning may be more effective [37]. The review concluded that further studies are needed on this topic, particularly involving people with multimorbidity in general, and across all age groups.

The present study has many strengths, such as the use of a whole-population sample, analyzed with the aid of strongly validated software, and the comprehensive data obtained on all diagnostic and specialist procedures, hospitalizations, drug consumptions, ED visits, and disregarding patients’ insurance status (given the universal health care coverage provided by the Italian NHS).

The main strength of our study lies in that it was population-based, thus minimizing selection bias, and independently collected data were used. The study also has several limitations, however, which need to be considered when interpreting the findings. First of all, the use of administrative data may mean that some conditions are under-represented [38]. There may not be enough data on functional status, for instance, or on chronic conditions with a high prevalence, such as visual or hearing impairment, or chronic pain—one of the most common conditions among patients ≥65 years old, and a significant burden on the management and economic resources of the health care system [39]. On the other hand, we included some health conditions—such as hypertension or dyslipidemia—which might be pharmacologically mismanaged in patients with multimorbidity, particularly in the elderly [8]. An approach that pays more attention to patient-centered measures and outcomes will be particularly important to decision-making for patients with multiple conditions as it will focus on outcomes that span conditions, and align treatments with common goals, which may also involve de-escalating treatments for one condition to optimize the treatment of another if this is more likely to achieve the patient’s goals [40].

We also tested for cluster multimorbidity by seeking any statistically significant associations between diseases, but we were unable to reveal any causal relationships between them, or any index diseases among them.

In conclusion, from a public health or health policy perspective, the growing burden of multimorbidity should make it very useful to estimate not only the most frequently occurring clusters, but also the most prevalent chronic conditions forming part of the most common patterns of multimorbidity, with a view to developing interventions to cope with the growing onslaught of the health demands of PCHCN.
